# Automatic detection of the carotid artery boundary on cross-sectional MR image sequences using a circle model guided dynamic programming

**DOI:** 10.1186/1475-925X-10-26

**Published:** 2011-04-11

**Authors:** Da-Chuan Cheng, Christian Billich, Shing-Hong Liu, Horst Brunner, Yi-Chen Qiu, Yu-Lin Shen, Hans Jürgen Brambs, Arno Schmidt-Trucksäss, Uwe HW Schütz

**Affiliations:** 1 Department of Biomedical Imaging and Radiological Science, China Medical University, Taiwan; 2 Department of Diagnostic and Interventional Radiology, University Hospital of Ulm, Germany; 3 Department of Computer Science and Information Engineering, Chaoyang University of Technology, Taiwan; 4 Institute of Exercise and Health Sciences, Sports Medicine, Vascular lab, University Basel, Switzerland

## Abstract

**Background:**

Systematic aerobe training has positive effects on the compliance of dedicated arterial walls. The adaptations of the arterial structure and function are associated with the blood flow-induced changes of the wall shear stress which induced vascular remodelling via nitric oxide delivered from the endothelial cell. In order to assess functional changes of the common carotid artery over time in these processes, a precise measurement technique is necessary. Before this study, a reliable, precise, and quick method to perform this work is not present.

**Methods:**

We propose a fully automated algorithm to analyze the cross-sectional area of the carotid artery in MR image sequences. It contains two phases: (1) position detection of the carotid artery, (2) accurate boundary identification of the carotid artery. In the first phase, we use intensity, area size and shape as features to discriminate the carotid artery from other tissues and vessels. In the second phase, the directional gradient, Hough transform, and circle model guided dynamic programming are used to identify the boundary accurately.

**Results:**

We test the system stability using contrast degraded images (contrast resolutions range from 50% to 90%). The unsigned error ranges from 2.86% ± 2.24% to 3.03% ± 2.40%. The test of noise degraded images (SNRs range from 16 to 20 dB) shows the unsigned error ranging from 2.63% ± 2.06% to 3.12% ± 2.11%. The test of raw images has an unsigned error 2.56% ± 2.10% compared to the manual tracings.

**Conclusions:**

We have proposed an automated system which is able to detect carotid artery cross sectional boundary in MRI sequences during heart cycles. The accuracy reaches 2.56% ± 2.10% compared to the manual tracings. The system is stable, reliable and results are reproducible.

## Background

Adaptations of the arterial structure and function were associated with blood flow-induced changes of wall shear stress which induced vascular remodelling via nitric oxide delivered from the endothelial cell [1]. In order to assess functional changes of the common carotid artery (CCA) over time, a precise measurement technique was necessary. In [2], only two static MR images representing the end-diastole and systole were taken for the measurement of the lowest and the highest arterial diameter during the heart cycle. However, this has been shown to have a higher variability than the measurement along the complete heart cycle. Furthermore the measurement on continuous images by hand-hold tracing was extremely time-consuming. Only a limited number of publications focused on the carotid arterial structure and function using MRI with advanced imaging technologies in healthy subjects were found [3-5]. The purpose of this study was to establish a novel automatic common carotid arterial wall detection algorithm in MRI sequences over several heart cycles in order to precisely determine carotid diastolic and systolic diameter changes along time and the CCA local compliance. For this purpose we have collected some MRI data from participants of the multistage ultra-aahn"Trans Europe Foot Race" in 2009 (TEFR09).

Regarding related researches on engineering aspect, the similar work to ours was found in [6]. The images they analyzed had plaques in the artery lumen. This was one additional problem they encountered more than ours. The other problems such as: contrast variations among blood, vessel wall and surrounding tissues, image artifacts caused by blood flow and random patient motion were similar to ours. Their system needed three user interactions: giving the system the artery's center point, a seed point inside the lipid core, and a circle surrounds the vessel. With the help of the prior knowledge combined with an elliptic model and fuzzy clustering, their system was able to identify the arterial boundary and plaque boundary.

Another previous study [7] was a measurement on arterial wall using discrete dynamic contour (DDC) [8]. Their method was semi-automatic because the system needed an initial contour of the inner wall contour. Moreover, their images were black blood vessel so that they were able to detect both the inner and outer wall boundaries of the carotid artery.

Another related article but focused on the coronary artery boundary detection was found in [9]. They proposed a surface-based method to detect the coronary artery boundary in the poor quality X-ray angiography based on a 3D generalized cylinder model. Since their application was on the X-ray angiogram, therefore, the proposed method was not suitable for the application on MRI sequences.

Our contributions are to develop an automatic method to measure the arterial boundary in MR images. It is able to detect the carotid artery center position in the first stage. In the second stage, the cross sectional arterial wall boundary can be detected via Hough transform and a circle model dynamic programming. The circle model dynamic programming lets the system control the output boundary to be somewhat round but having the ability to detect the fine structure.

The paper is organized as follows. The image sources and MRI protocol are introduced in Section 2. Section 2.1-2.2 describes how to detect the artery lumen center position. In Section 2.3, the circle model guided dynamic programming is issued in details to solve our problems. Afterwards, results are given in Section 3. We then discuss properties of the proposed scheme in Section 4. Finally the conclusion is given in Section 5.

## Methods

The MR-measurement of the maximal systolic and distal vessel-diameter of the CCA with additional blood pressure information leaded to local compliance of the arterial wall. After the approval of the local ethics committee in accordance to the Declaration of Helsinki, 12 participants in the TEFR09-project of the German Research Foundation (DFG-Project GZ: SCHU 2514/1-1, AOBJ: 565344) have been collected for vascular studies based on MR image sequences. One MRI sequence of one subject was randomly chosen from these 12 subjects for the validation of the novel detection algorithm of the CCA lumen presented in this study.

To acquire the vascular MRI sequences, a mobile 1.5-T MR imager (Siemens - Avanto™, Model Mob. MRI 02.05, Siemens Ltd., Erlangen, Germany) and a custom-designed four-channel phased dual mode neck matrix coil with 4 integrated preamplifiers (Siemens Ltd.) were used. The movement artifact was minimized via using a dedicated head restraint system (head coil, Siemens Ltd.) to fix the head and neck in a comfortable position (patient position: supine, head to feet).

After an initial coronal localizer, three fast localizers (triplanar TRUFI: "true fast imaging with steady state precision"; Siemens Ltd.) were used to identify the axial perpendicular acquisition location at the right CCA 10 mm inferior the carotid bifurcation. Contrast media could not be used in this study because the athletes did not accept it.

To increase the spatial resolution of the measurement (cross section view of CCA) a T2*-weighted gradient-spoiled gradient-echo cine-sequence (FLASH: "fast low angle shot", Siemens Ltd.) with prospective two dimensional ECG gating (cardiac triggering) was used. Parameters were set to be: flip angle 15°, echo time 5.41 ms, repetition time 34.74 ms, slice thickness 6 mm, field of view 289 cm^2^, matrix size 320×320, pixel size 0.53125 mm ISO, pixel bandwidth 250, number of images per sequence: 50 images for one RR-cycle. The imaging acquisition time was approximately 5 minutes for each sequence.

### 2.1 Carotid artery position detection

The carotid artery position detection is the first procedure because it can reduce the following computation time. This procedure detects only the rough artery's center position but not the artery boundary. Here we propose an easily implemented but fast algorithm to perform this work.

**Figure 1:**
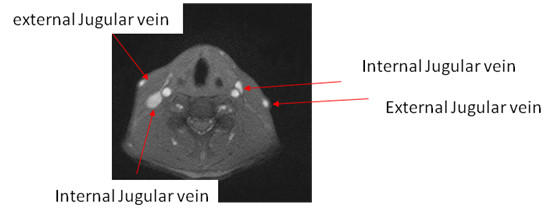
**One of the MR images contains the carotid arteries we interest**. The carotid arteries are the ones near internal jugular veins which are round having a lighter intensity.

To identify the carotid artery we firstly analyze its features. Normally some large vessels can be seen in MR images including carotid arteries, internal jugular veins, and external jugular veins (see Figure 1). Among them two most largest vessels are carotid artery and internal jugular vein. The internal jugular vein is often larger than the carotid artery, but only in a supine position because of the filling at a lower pressure. Another exception is having an abnormal hypoplastic situation. This is however not the case in our subjects. The other differentiation is that the cross-sectional view on the common carotid is always round with the exception of plaques in the artery lumen. Moreover, the vessel lumen in MR images has a larger intensity. This is because blood in vessels shows higher signal intensity in the T2-weighted FLASH-sequences. Due to the blood flow there is also a phase shifting. These are reasons for a higher intensity of vessel lumen in contrast to dark vessel wall. These three features (area size, shape, and intensity) are useful information in the identification of the carotid artery.

The first feature we utilize is the intensity to identify vessel lumen from other tissues. The image contains foreground (the subject) and background. The background is the dark area surrounding the subject. The foreground contains mussels, vessels, bones, air chambers and other tissues or organs. Among them the air chamber is dark so it is easily to be excluded like the background. The rest is to identify vessels from other tissues. Since the vessel lumens are lighter than the other, we are able to classify them using the intensity as a feature. Let R denote the raw image. The Otsu's thresholding technique [10] is applied in two stages. The first stage is to segment the foreground (excluding the air chambers) out of the image. The extracted foreground is then segmented via Otsu's technique again in the second stage. After this process some vessel lumens are able to be segmented, however, with some noises in it. The first stage can be formulated as follows.(1)

where T_1 _is the threshold obtained by Otsu's method. The background is marked by -1 which will be ignored in the second stage. The second stage is formulated as follows.(2)

where T_2 _is the threshold obtained by Otsu's method which is a value between the gray value of the vessel lumen and other tissues. Notably the computation of T_2 _is based on the precondition of ignoring the background marked as -1 in the first stage. After this process vessel lumens are segmented out.

The resultant image contains noises needed to be removed. The binary morphological opening operation with a structure element is then applied to filter the noise and cut possible connections between the internal jugular vein and the carotid artery. The filtration is formulated as(3)

where 's' is the structure element with a disk shape (radius is 1). More clearly, it has a central pixel (the reference point) and the four-neighborhood pixels.

Afterwards, the rest features (area size and shape) are utilized to identify the carotid artery from other vessels. This process is divided into left and right parts. Assume we are processing one part of them, two largest areas are chosen and they are the internal jugular vein and the carotid artery. Their boundary points are obtained by using a simple Sobel operation [11]. Afterwards, the PCA (principal component analysis [12]) is applied to get the major axis and minor axis. The length ratio of these two axes is a feature to indicate if the shape is round. Via this scheme, we are able to identify the carotid artery from other vessels. Once its center position is estimated, a region of interest (ROI) denoted as *R*_s _is extracted from the source image *R *to the following procedures while artery center centered at *R*_s_. The size of region (*R*_s_) depends on the pixel size of MR images and the anatomic knowledge how large the carotid artery can be. This can be calculated in prior.

### 2.2 Carotid artery boundary detection

#### 2.2.1 Directional gradient

The results obtained by the method addressed in Section 2.1 do not offer accurate artery boundaries. This is because the Otsu's thresholding technique never offers good results in case that the intensity is not consistent for each object (here the carotid artery) to be measured. Especially it is possible that the morphological opening operation shrinks the artery's actual size. Therefore, we propose a method to detect the accurate artery boundary. Since the artery boundary has intensity different to its surrounding area, the gray level gradient is useful information. However, the internal jugular vein is very close to the artery in images so that it makes the boundary detection difficult if we consider only the intensity gradient only. This case is worse if the intensity gradient on the vein boundary is stronger than that on the artery boundary. We therefore take the direction into consideration and name this gradient to be directional gradient. In literatures we do not find any similar publication except the directional gradient vector flow in [13]. Our consideration is that: since the artery lumen is brighter than its surrounding areas, gradients resulted from bright pixels to dark pixels are of interest. If the artery center is known, then the directional gradient can be found that is parallel to the radiation lines centered from the artery. Actually the directional gradient is a special case of multi-directional gradient.

**Figure 2:**
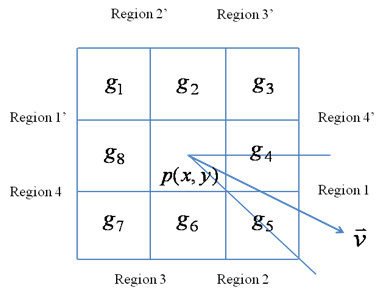
**The geometric definition for calculating the directional gradient**.

Figure 2 depicts the geometric construction of a directional gradient. Consider the point we are processing is *p*(*x*, *y*) ∈ *R*_s_, a unit vector () connecting *p *and the artery center point is computed. The whole space where  might be located is divided into eight regions, i.e. the angle resolution is 45 degrees. The gray levels surrounding *p*(*x*, *y*) are denoted as from *g*_1 _to *g*_8_. Since the y-axis in image is from top to down, we define Region 1 to be the area where , where θ is the angle between  and the x-axis, (-π ≤ θ ≤ *π *). If  is located in Region 1, the calculation of the directional gradient considers only two gray-level pairs (*g*_4_, *g*_5_) and (*g*_8_, *g*_1_) as follows:(4a)

where *t *= 4*θ*/*π *. If  is located in Region 1' (-π ≤ θ ≤ - 3π/4), the formula can be rewritten as:(4b)

Where *t *= 4(*θ *+ *π*)/*π*. The calculations when  is located in other regions are similar. Via this scheme an edge map representing the gradient intensity can be obtained and denoted as *R*_e_. Notably, we are looking for gradients resulted from bright pixels to dark pixels. Therefore, the edge map we look for is a minimal value. The negative gradient denotes the boundary gray level changing from bright pixel to dark pixel along vector  which is we want. Via this way, the positive gradient resulted from the jugular vein very close to carotid artery will not affect the searching of the artery boundary. Afterwards, positive values in *R*_e _are set to zeros and the Otsu's thresholding technique is applied again to find a threshold value. The binarisation technique is applied to *R*_e_: values under the threshold are set to ones. The resultant binary image is denoted as *R*_b_.

### 2.3 Circle model and dynamic programming

The round shape information of artery is important. It is used to avoid possible errors caused by local noises. These errors include the heterogeneous gradient obtained in the artery lumen and at the boundary. To alleviate this problem we apply Hough transform [14] to detect round objects in *R*_b_. The Hough transform is a feature extraction technique used in image analysis, computer vision, and digital image processing. There are three variables to be determined, i.e., the circle center position (*x *and *y *coordinates) and the radius. Although the radius of artery is unknown in advance, its range of variance can be estimated. Therefore, we have to calculate its Hough transform by varying the radius from *r*_1 _to *r*_n _∈ N. After transformations each radius obtains an accumulator matrix. We search the maximum value in each accumulator matrix and find out the one which has the maximum value among all accumulator matrices. The corresponding radius and position is the artery's radius and center position, respectively.

Although the artery lumen is in general round, however, it is not the case from the pixel's view point. In addition, not all artery lumen is pure round during the heart beat cycle. Some of them are elliptic. A fine tuning is then necessary to obtain a sub-pixel accuracy. For this reason, we propose a method to identify the artery boundary based on a circle model.

Dynamic programming is a method of solving complex problems by breaking them down into simpler steps used in mathematics and computer science [15]. It is applicable in image processing to solve optimal problems such as finding a minimum (or maximum) with some given constraints[16,17]. However, the limitation of using this technique in images is that it cannot solve the closed form contour. One solution is to transform the image from Cartesian coordinate to polar coordinate [18] and then apply dynamic programming on the polar coordinate. Note that this procedure applies only on a region of interest (ROI). However, two preconditions have to be satisfied: 1) the rough center position is known; 2) the contour has a star-like shape. Our problem meets these two preconditions.

The dynamic programming is issued in details as follows. In Section 2.2.1 we have obtained the directional gradient *R*_e_. Let *M *and *N *denote the number of rows and columns of *R*_e_, respectively. Normalization is applied on *R*_e _so that values range from to -1 to 1, i.e. -1 ≤ *R_e _*≤ 1. Since the center of *R*_e _is the artery center, we transform *R*_e _to polar representation and denote it as *R*_p_. The x-axis of *R*_p _represents angle 0 ≤ θ ≤ 2π and the y-axis represents the distance to the center point in *R*_e_. Notably, θ = 2π represents the start point copied to the end of the matrix *R*_p _to convince the continuity between the start and end point. Dynamic programming is then searching a curve from left to right *R*_p _in which represents the artery boundary. Some features are taken into considerations.

1) Curve continuity: A variable for continuity is considered. Let *d_r _*denote the maximal range that nodes in column *x - *1 are allowed to jump onto the next column *x *in either up or down directions. Therefore, each node has maximum (2*d_r _*+ 1) possible link paths to its previous column. If *d_r _*is set larger, the curve's roughness and the computation time are both increased. The smoothness of the curve is quantified by *j *= *y*_*x*-1 _- *y*_*x *_which is embedded into the cost function.

2) Circle model: The circle model having a known radius is embedded into the structure to guide the dynamic programming. This is based on the fact that the artery boundary is near round and the radius is estimated by Hough transform in prior. A Gaussian model is used to generate the strength how strong the dynamic programming is guided by the circle model. Let *r *denote the known circle radius, the strength is formulated as , where *σ *is a variable controlling the strength of guide. If *σ *is getting smaller, the Gaussian has a thin but sharp shape and the circle model has a larger effect on the result, i.e. it is a more circle-like boundary. If *σ *is getting larger, the Gaussian term vanishes and it works like a normal dynamic programming without the circle model. Since *y *and *r *are both integers, a look-up table can be set to reduce the computation time.

3) Directional gradient: The directional gradients are very important information to detect the artery boundary accurately. Gradients having negative values denote the carotid artery boundary, while positive gradients denote other boundary which we treat them as noises. Thus, the boundary detection problem is then transformed to an optimization problem which searches an optimal contour:

 subject to some constraints, where *p_i _*is the point on the *i*-th column in the matrix *R_p_*, and *p_k _*and *p*_*k*+1 _are neighborhood. This optimization function can be reformulated to be suitable for implementing dynamic programming with respect to a cost function formulated as follows.(5)

subject to 2 ≤ *x *≤ *N*, 1 ≤ *y *≤ *M*;

where *α *is a weighting parameter. The *C*(*x*, *y*) is a two-dimensional cost map. The global optimization problem is the same to its sub-problem *C*(*x *- 1, *y*), *C*(*x *- 2, *y*), and vice versa. We set *C*(1, *y*) = *R*_p_(1, *y*) to be a boundary condition. If *d_r _*= 1, the optimal index j* can be determined by the following equation:(6)

Therefore, the index can be stored in the coordinate matrix *X*(*x*, *y*) = *y *+ j*. In this construction, small cost values indicate higher likely boundary information. The position with the minimum cost value in the cost map *C*(*x*, *y*) is searched. With a backward search from N to 1 in *X*, the complete coordinates (*p*_1_*p*_2_*p*_3_...*p_N _*) of the artery boundary can be determined, which is the optimal solution to this problem. Notably, the processes addressed in Section 2.3 are applied only on the extracted ROI (*R*_s_, *R*_e_, and *R*_p_) to reduce the computation time tremendously. Results obtained by dynamic programming are integers. A moving average technique [19] is applied to make the boundary smoother. In order to reduce the computation time, we simply average the neighbouring 4 points and the center point. The resultant polar coordinates are then transformed back to Cartesian coordinates.

### 2.4 System reliability

The proposed system has been studied for the reliability against different noise levels and different contrasts.

In order to study the effect on different contrasts, we test one image sequence with different contrasts. Let [0, g_max_] be the contrast resolution of the raw image. The contrast is degraded by a ratio ranging from 0.9 to 0.5, in a step of -0.1, and we name them to be 90% contrast to 50% contrast, respectively. Therefore, the contrast resolution of the 50% contrast will be [0, int(0.5 g_max_)] and vice versa, where int(x) converts a number 'x' to be an integer closest to 'x' . All raw images are converted to degraded images based on a given ratio. Thereafter, the proposed system is applied on these contrast degraded images and the carotid artery cross-sectional lumen area of each image is calculated for the following comparison. The comparison is performed by calculating their relative signed errors as follows:(7)

where A_Automated _(*i*) and A_Manual _(*i*) are areas calculated by the automated and the manual drawing on image number *i*, respectively.

To study the system reliability against noise levels, we add artificial white noises (randomly generated) with a given SNR (signal-to-noise ratio) ranging from 20 dB to 16 dB with a step of -1. The SNR calculation is given as follows:(8)

where M × N denotes the image dimension, g (*x*, *y*) and *n*(*x, y*) are the intensities of image and noise at image coordinate (*x, y*), respectively. The MR image at (*x, y*) has an intensity g(*x*, *y*)≥0 and the noise intensity might be negative. To calculate the SNR we define  if *g *(*x*, *y*) = 0 or *n *(*x*, *y*) = 0.

## Results

**Figure 3:**
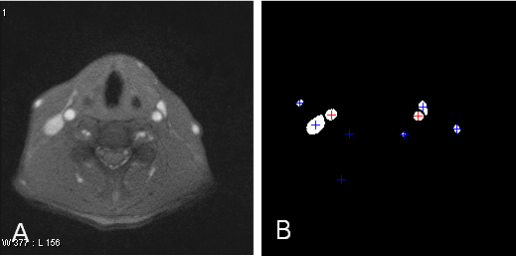
**Results of carotid artery position detection**. (a) The raw image. (b) The center position of each detected vessel lumens are marked by '+'. The carotid artery (denoted as arrows) is recognized by its shape and size features.

Figure 3 shows results of the first process: carotid artery position detection. The two stages Otsu's thresholding technique can lead to some noises which are removed by morphological opening operations as shown in Figure 3(b). Afterwards, the round shape and area size are used as features to distinguish carotid arteries from those vessel lumens. Their corresponding artery centers can be calculated which are used as reference points in calculating directional gradients.

**Figure 4:**
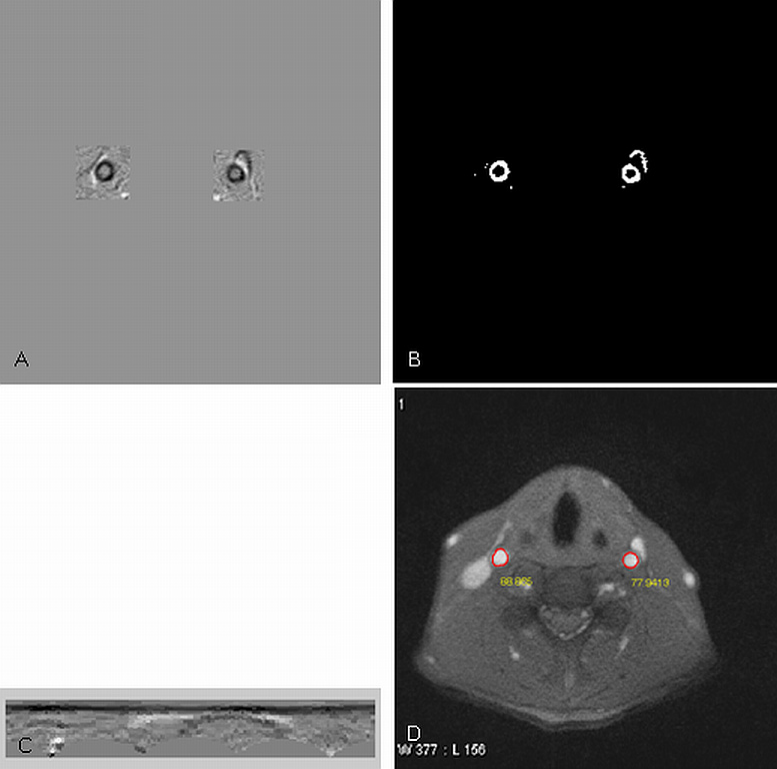
**Results of the carotid artery boundary detection**. A. Results of directional gradients. B. Results of using Otsu's thresholding technique. C. The ROI (*R*_e_) is transformed to the polar representation (*R*_p_)for dynamic programming. D. Results of circle model guided dynamic programming. (*α *= 0.3, *σ *= 2, and *d_r _*= 1).

The directional gradient is computed on a ROI (*R*_s_) and the normalized result (*R*_e_) is shown in Figure 4(a). The surrounding gray area having 0 values are not calculated. The dark area represents negative gradients we need whereas the white area denotes positive gradient which are noises. Since the positive gradients are very close to the negative gradients, if the gradient direction is not considered it is almost impossible to distinguish them. Figure 4(b) shows the binary result (*R*_b_) using a thresholding technique for Hough transform. The Hough transform can determine a radius which will guide the dynamic programming in detecting the artery lumen boundary. Figure 4(c) is the polar representation of *R*_e _on the left-hand side (right carotid artery). The dynamic programming searches a curve from left to right which minimizes the given cost function defined in equation (5).

The detected artery lumen center position is used to predict the center in the next image. Similarly, the detected artery lumen radius is used to set the size of ROI in the next image. Here we expend 1.5 times radius from the center to each side (left, right, up, and down) to define the size of ROI. For the reason of explanation, the ROIs shown in Figure 4 are larger than 1.5 times.

**Figure 5:**
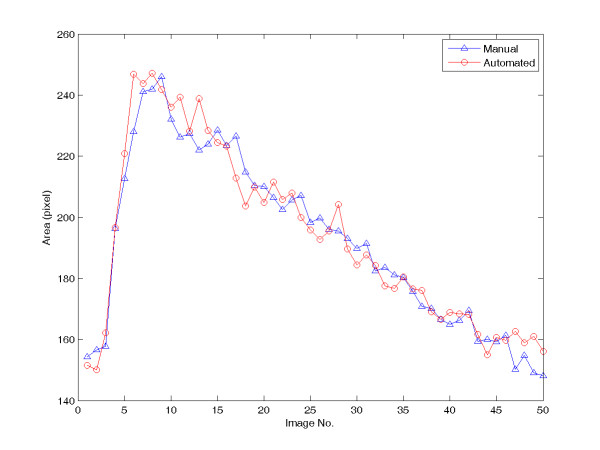
**The comparison of automated and manual results of the right carotid artery**. (1 pixel size = 0.28 mm^2^).

In order to explore the accuracy of the proposed automated system, the accuracy control is necessary. For this purpose, an image sequence containing 50 images is used to compare the manual boundary tracing and the automated identification. The right carotid artery is chose to compare. The areas are calculated and shown in Figure 5. The averaged relative error is 2.56% and its standard deviation is 2.10%. The averaged relative unsigned error is defined as follows:(9)

where ε_i _has been defined in equation (7), N = 50.

**Figure 6:**
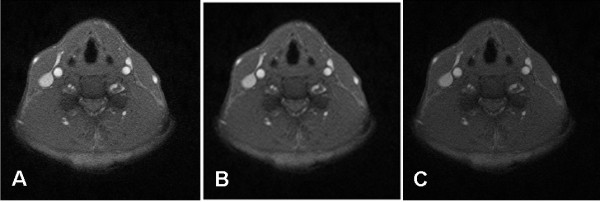
**Three images with different contrast degradations**. A. The raw image. B. A 70% contrast degraded image. C. A 50% contrast degraded image.

**Figure 7:**
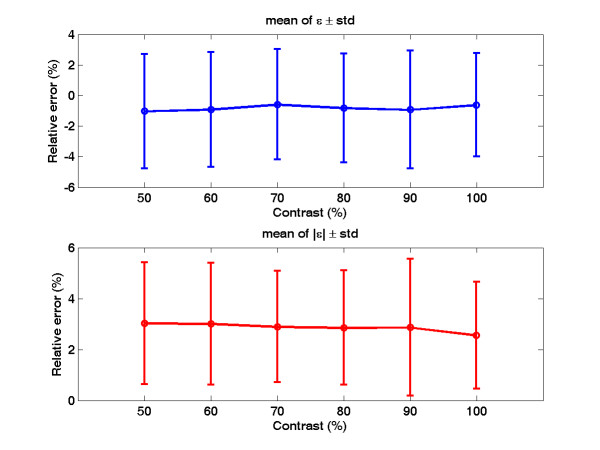
**Comparison (signed and unsigned relative error) between the manual drawing and the automated method on different contrast degraded images (from 50% to 100% contrast)**.

**Figure 8:**
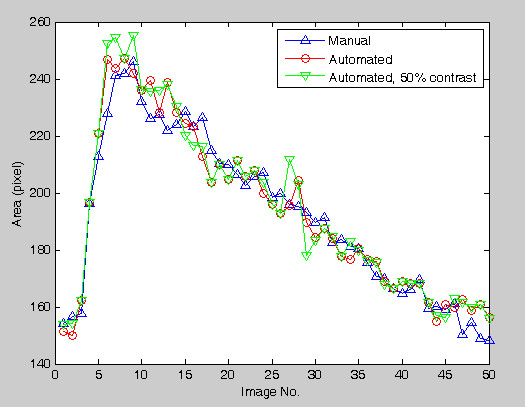
**The areas computations of each image are shown**. The line having circles is made from 100% contrast images, i.e., without the contrast degradation. The line having triangle (down) is made from 50% contrast images. The line having triangle (up) is made by manual drawing.

To investigate the system reliability with respect to different contrast resolutions, the images are degraded. Figure 6 shows the raw image and two degraded images with 70% and 50% contrast. The proposed algorithm is repeated on the degraded images and the comparison results are shown in Figure 7. In Figure 7, different contrast resolutions do not show significant differences (signed error ranges from -0.58% ± 3.6% to -1.03% ± 3.75%, unsigned error ranges from 2.56% ± 2.10% to 3.03% ± 2.40% ) in calculating the carotid artery cross-sectional areas. The relative unsigned averaged errors are almost consistent in different contrast resolutions. The signed error shows that the automated method produces a larger area than the manual tracing does. However, this bias is very limited. Figure 8 shows the comparison plot of areas computation with respect to image number with different conditions. The line having triangle (up) is the manual drawing. Two automated results made from 100% and 50% contrast images are superimposed on the result of manual drawing to show the difference. From the plot there are only limited errors between them. The experiment of 50% contrast has the largest unsigned error (3.03% ± 2.40%).

**Figure 9:**
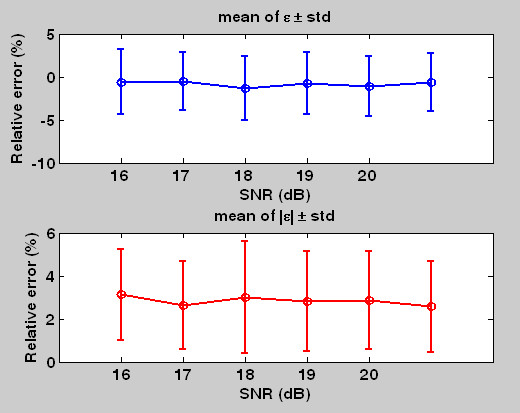
**The comparison (signed and unsigned relative error) between manual drawing and automated method applied on different noise degraded images**. The noise level is represented by SNR (dB). The 20 dB means the signal intensity is 10 times the noise intensity. The right one without SNR is the raw image (without artificial noises).

Figure 9 shows the comparison (relative error) with respect to different noise levels. The image having less SNR has larger noise level. From the result we see a tendency of increasing relative error when the SNR is decreasing. The signed relative error ranges from -0.53 ± 3.77% to - 1.13% ± 3.49% whereas the unsigned relative error ranges from 2.56% ± 2.10% to 3.12% ± 2.11% . There is no significant difference between different noise levels ranging from 16 to 20 dB (SNR).

**Figure 10:**
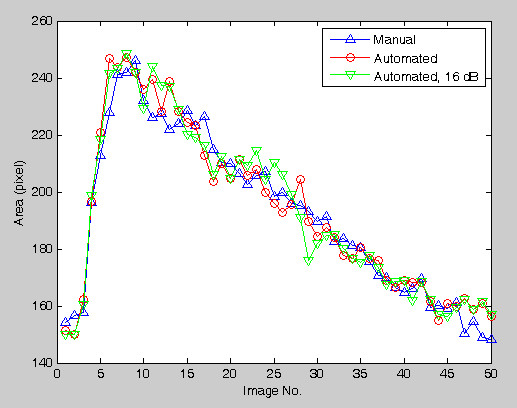
**The areas computations of each image are shown**. The line having circles is made from raw images, i.e., without the artificial noises. The line having triangle (down) is made from SNR = 16 dB noise-degraded images. The line having triangle (up) is made by manual drawing.

To compare the error on each image number, the results made by the automated method applied on raw images and images having 16 dB SNR are superimposed on the manual drawing result. Each error is very limited and there is no abrupt large error among them (Figure 10).

**Figure 11:**
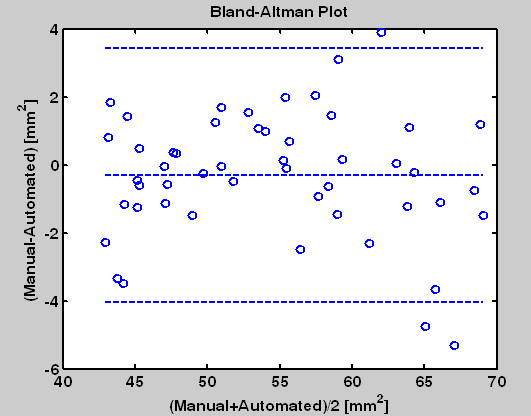
**The Bland-Altman plot is used to compare the manual tracing and the automatic identification results**. The middle line is the average. The upper and lower lines denote ± 2 standard deviations.

To summerize the system reliability we use the Bland-Altman plot as shown in Figure 11. The mean of the two measurements (manual drawing and automated method) are assigned as the abscissa (x-axis); the differences between the two measurements are assigned as the ordinate. From the result we see their differences are mostly under 4 mm^2^.

The computer system has Intel^® ^Core™ 2CPU T5600, 1.83 GHz, with 2 GB RAM. The programs are based on the Matlab platform [20]. The computation time for 50 images processing is around 30 seconds. More results are downloadable under our website.

## Discussion

In this study we use a circle model in Hough transform and the dynamic programming instead of the ellipse Hough transform because of the consideration on the computation time. Full ellipse detection is rarely performed because it is very slow. It requires a 5 dimensional parameter space - as opposed to 2 for straight line detection and 3 for circle detection. Although the gradient direction can be taken into consideration to reduce the computation time [21], it still needs much more time than that in our design. Moreover, the artery's shape can be changed if a plaque exists. Our design has the advantage to detect boundary which is not a circle or an oval.

Since our algorithm uses area, shape, and intensity as features to identify the carotid artery position, the conditions (prerequisites) that the carotid artery is able to be identified are: 1) there are less or no plaques in the artery; 2) the blood flow maintains in a level so that the intensity in artery lumen in MRI is distinguishable from neighbouring tissues. Our subjects are healthy runners, although some are old people, there are less plaques in the artery lumen. Therefore all carotid artery lumens can be modelled by a circle model. The blood flow in the carotid artery is different from that in the femoral artery. It does maintain at a level so that the intensity in the lumen is distinguishable from the artery wall and other tissue nearby. Therefore, there is no problem to identify the carotid artery centers using our proposed algorithm.

Regarding the chosen of parameters, there are three parameters in our scheme: *α*, *d_r_*, and *σ *. Normally, the discontinuity is prevented by setting *d_r _*= 1. We suggest the range of *α *to be (0,1]. If *α *becomes smaller, the output curve is more rough. On the contrary, the curve becomes smooth if *α *is set near to 1. The standard deviation *σ *is a control by the circle model. The range we suggest is [0.4, 4]. If *σ *≤ 0.5, then the output curve is nearly round. If *σ *≥ 1 then it is able to detect the fine structure such as plaques in the artery lumen.

Based on the study on different contrast resolutions (from 50% to 100%) and different noise levels (SNR ranges from 16 dB to 20 dB), the proposed method has shown its robustness and reliability against contrast resolution and noise.

## Conclusions

In conclusion, we have proposed a fast and robust scheme to detect the carotid artery boundary in MR image sequences fully automatically. This scheme is divided into two stages: (1) detect the center of the carotid artery (2) detect the boundary of the carotid artery. We combine the circle model with the dynamic programming so that the resultant boundary is circle-like shape. The accuracy control shows that the averaged relative error of the automated results compared to the manual results is 2.56% and the standard deviation is 2.10%. Via this system we are able to analyze tremendous amount of images and all results are repeatable.

## Competing interests

The authors declare that they have no competing interests.

## Authors' contributions

This work can be divided into two parts: medical and engineering aspects. HB, HJB, AST and US are responsible for the scientific planning of medical background. CB and SHL are responsible for data processing. The manual drawings are under the supervision of US. DCC are responsible for the development of image processing techniques and programming. The programming codes are Matlab codes. YCQ and YLS have made some contributions on coding. The manuscript is written by DCC, AST and US. All authors have read and approved the manuscript.
